# Posterior Auricular Perichondrial Cutaneous Graft Combined With Cartilage Strip in Nostril Reconstruction

**Published:** 2008-08-20

**Authors:** DW Schmid, PG Di Summa, R Wettstein, P Erba, W Raffoul, DF Kalbermatten

**Affiliations:** ^a^ Department of Plastic, Reconstructive and Aesthetic Surgery, University of Basel, Basel, Switzerland; ^b^ Department of Plastic, Reconstructive and Aesthetic Surgery, University Lausanne, Lausanne, Switzerland

## Abstract

**Objective:** Reconstruction of alar structures of the nose remains difficult. The result has to be not only functional but also aesthetic. Different solutions to reconstruct alar defects are feasible. A good result that meets the specific demands on stability, aesthetics, and stable architecture without shrinkage of the area is not easily achieved. **Method:** A perichondrial cutaneous graft (PCCG), a graft consisting of a perichondral layer, fatty tissue, and skin that is harvested retroauriculary, is combined with an attached cartilage strip. **Case Result:** A 72-year-old patient suffering from basal cell carcinoma of the ala of the nose underwent the reconstructive procedure with a good result in 1 year in terms of stability, color match, and graft take. **Conclusion:** First, a strip of cartilage had been included in a PCCG where tumor resection required sacrifice of more than 50% of the alar rim. The case shows that one can consider a cartilage strip–enhanced PCCG graft to reconstruct alar defects.

Defects of the lower third of the nose that involve the rim are challenging. A “misplaced patch” appearance, depressed scarring, and nostril retraction should be avoided and nasal airway patency and an esthetically pleasant nostril shape should be maintained.^1^

The perichondrial cutaneous graft (PCCG), first described by Brent and Ott in 1978,^2^ is a composite graft of skin and perichondrium harvested from the anterior conchal bowl of the ear. This composite graft, consisting of epidermis, dermis, a small amount of subcutaneous tissue, and a perichondrial vascular plexus in the underlying perichondrium,^3^ has a naturally curved contour and yields excellent cosmetic and functional results in reconstruction of the nasal tip and ala and also in auricular and periorbital defects. Portuese et al^4^ showed that PCCGs contract less and maintain the original thickness if compared with skin grafts, secondary to improved (re)vascularization through the perichondrial vascular plexus, thereby increasing the chance for graft take, even in case of suboptimal graft bed.^3^ Moreover, it has been shown that the outer perichondrial layer included in the graft rapidly induces fibrous growth and provides stable connections to the wound bed^5^ resulting in symmetric stability without inspiratory collapse after reconstruction.^6^

Harvesting of the PCCG from the posterior auricular donor site instead of the anterior conchal bowl has simplified the procedure, while maintaining excellent functional and aesthetic outcomes.^6^ The posterior conchal donor site has a low, long-term-complication rate and a high patient satisfaction with psychological abstraction because of the concealed donor site. For these reasons the PCCG grafting procedure is a simple and reliable option, with minimal donor-site morbidity and a low complication rate, that can be performed as a single-stage outpatient procedure leading to superior results when compared with full-thickness skin grafts (FTSGs).^3^

We report a case treated with a PCCG graft from the posterior conchal region that was combined with a full-thickness strip of cartilage for reconstruction of a large alar and nostril rim defect. This combination resulted in excellent alar stability and contour because of the cartilage support and resulted in an optimal wound healing, because of the previously mentioned properties of graft revascularization and survival for the perichondrial layer.

## CASE REPORT

**Figure 1:**
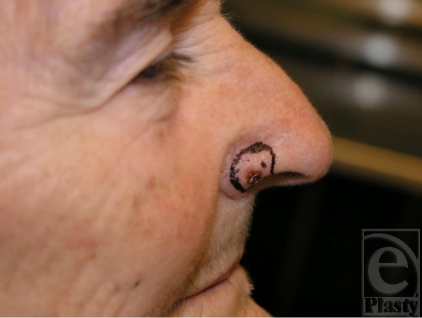
Patient before resection of basal cell carcinoma of ala of the right side.

**Figure 2:**
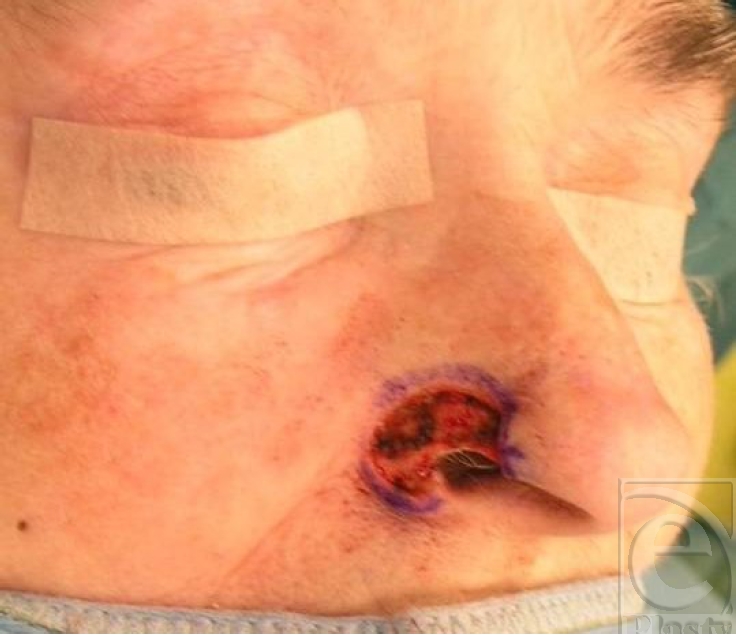
Resection of tumor revealing a hemialadefect.

**Figure 3:**
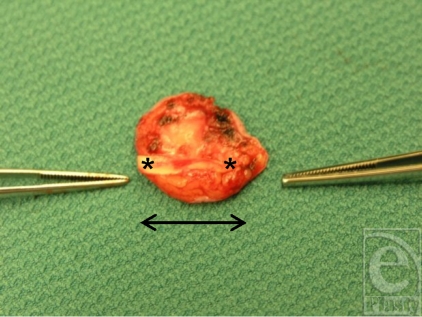
Perichondral graft harvested from retroauricular. *Cartilage strip attached, the arrow shows orientation.

**Figure 4:**
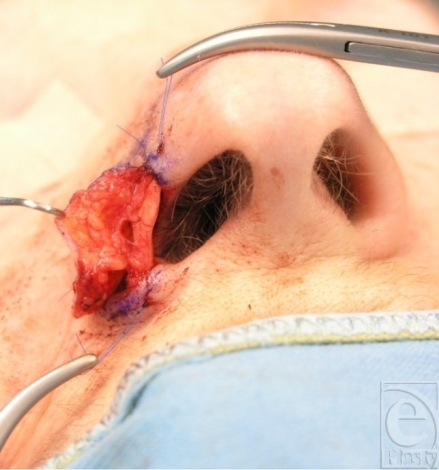
Setting in of the graft with focus on the cartilage strip that creates the lower rim.

**Figure 5:**
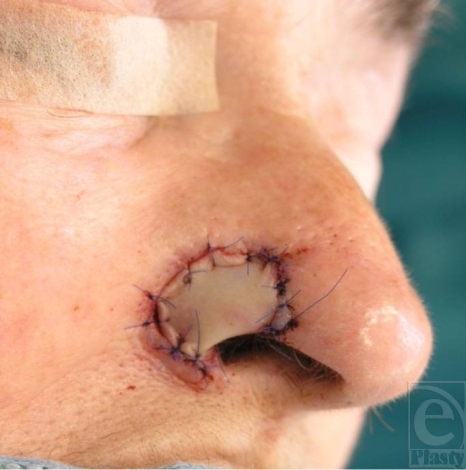
Perichondral graft applied on the nostril defect.

A 72-year-old female patient presenting with morphoeic basal cell carcinoma of the right ala underwent 1-stage tumor resection and defect reconstruction (Fig 1). The defect measured 13 mm × 16 mm and included about 50% of the right alar rim (Fig 2). For defect reconstruction, a PCCG including a strip of cartilage was harvested from the posterior auricular surface (Fig 3). To prevent alar rim notching, the cartilage strip was set in a pocket created into the intact portion of the alar rim (Figs 4 and 5). The donor site was closed with a local flap and it healed by primary intention.

**Figure 6:**
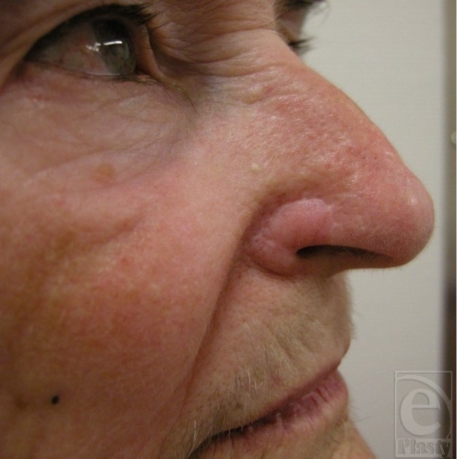
Zoomed view of the result after 1 year.

**Figure 7:**
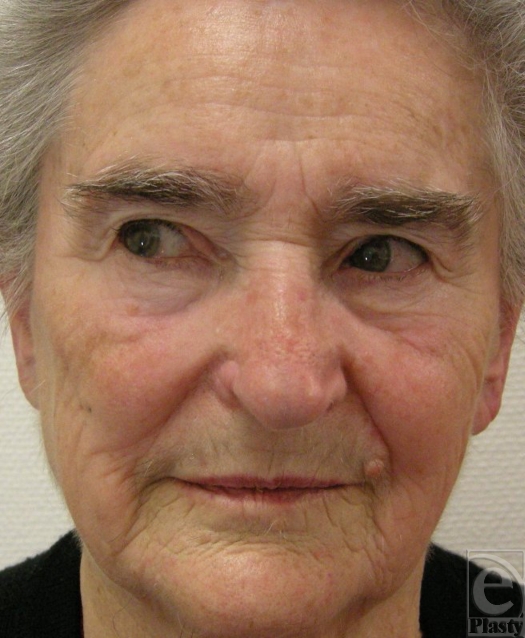
Complete frontal view of the patient (post–1 year).

**Figure 8:**
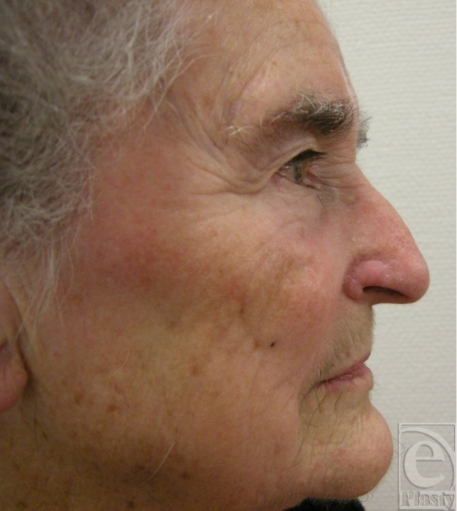
Complete lateral right view of the patient with graft tissue in site (post–1 year).

**Figure 9:**
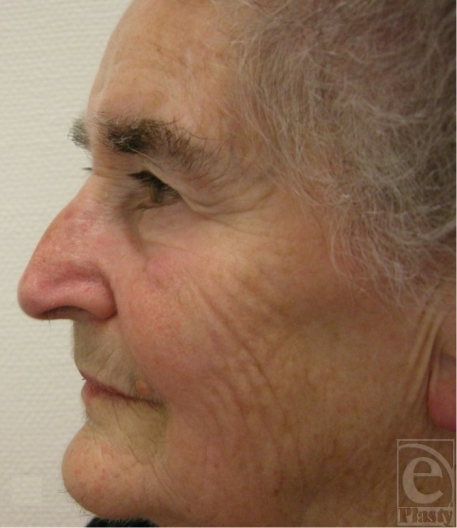
Complete lateral left view of the patient, as control.

Postoperative course was uneventful with primary healing and a 100% graft uptake. Follow-up after 1 year showed a nice alar rim contour with barely noticeable notching at the posterior border to the alar base (Figs 6–9). Although the graft itself had an excellent color match and was only slightly contracted, the scars still showed some signs of activity.

## DISCUSSION

Depending on size and site, different reconstructive options, such as primary closure, FTSG, local flaps, and composite grafts are available to cover defects of the lower third of the nose. Because the skin is often thick, sebaceous, and particularly stiff,^1^,^7^ an elliptic incision with primary closure yields poor results with a depressed scar, which can considerably contract and distort the topography of the aesthetic unit.^7^ Therefore closure with FTSGs and local flaps are mostly employed. However, in the lower third of the nose, cosmetic appearance of FTSGs is often suboptimal in terms of thickness, rim contour with scar contraction and notching, color match, and poor graft survival over the exposed cartilage.^7^,^3^ When compared with FTSGs, local flaps are subject only to minimal contraction and provide a better color match; furthermore, flaps have higher survival rates than grafts.^3^ Despite these advantages, local flaps are associated with extra incisions that cross aesthetic subunits,^1^,^3^ and are delicate in terms of thickness, notching, and tip distortion by flap rotation or transposition because of the relative mobility of the nasal tip. The human eye can easily detect any difference in nostril rim contour after flap surgery despite the best design.^1^ Results with flap coverage are frequently less than satisfactory, requiring secondary debulking and scar revision procedures.^1^,^7^

Composite skin grafts for nostril rim reconstruction, which include epidermis, dermis, superficial layers of subcutaneous tissue, fat, or cartilage segments, have been extensively described in the literature.^1^,^7^,^8^ Particularly, composite chondrocutaneous auricular grafts harvested from the conchal cavity^9^ or the helix can provide the required thickness for both inner nasal lining and nostril rim.^8^,^10^ Even if providing good cosmetic outcomes when placed over cartilage in the distal portion of the nose, their survival, depending on revascularization from recipient bed and defect periphery, might be critical.^1^,^10^ To improve vascularization, coverage by skin flaps,^10^ attached dermal pedicles,^11^ and modified composite grafts have been described.

Encouraged by the results obtained with PCCGs in terms of stability, color match, and graft take, we modified the graft for this case that involved about 50% of the alar rim. Nevertheless, PCCGs are limited in size and are better sized below 20 mm in diameter; therefore, we did not resect the whole aesthetic unit of the ala but dissected only the posterior part that is important for the stability of the nostril and where the tumor site was. The inclusion of a small strip of cartilage did not affect graft take in contrast to our previous experiences with composite grafts, including cartilage for nostril rim reconstruction, usually harvested from the base of the helix. The stability provided by the PCCG in combination with the strip of cartilage used mainly to prevent notching and collapsing was sufficient to withstand forced inspiration. The caudal part of the PCCG overlapping the cartilage strip could be nicely contoured to reconstruct the alar rim and the lateral cartilage strip extension was positioned into a small pocket of the intact alar rim to prevent retraction resulting in alar notching.

In conclusion, this case of a PCCG from the posterior auricular surface including a strip of cartilage resulted in a nicely contoured reconstruction of the alar rim. In our case this modified composite graft was superior in graft take and alignment to previously described grafts that include a higher percentage of full thickness cartilage.

## References

[1] Hubbard TJ (2004). Leave the fat, skip the bolster: thinking outside the box in lower third nasal reconstruction. Plast Reconstr Surg.

[2] Brent B, Ott R (1978). Perichondro-cutaneous graft. Plast Reconstr Surg.

[3] Gloster HM, Brodland DG (1997). The use of perichondrial cutaneous grafts to repair defects of the lower third of the nose. Br J Dermatol.

[4] Portuese W, Stucker F, Grafton W, Shockley W, Gage-White L (1989). Perichondrial cutaneous graft. An alternative in composite skin grafting. Arch Otolaryngol Head Neck Surg.

[5] Duynstee ML, Verwoerd-Verhoef HL, Verwoerd CD, Van Osch GJ (2002). The dual role of perichondrium in cartilage wound healing. Plast Reconstr Surg.

[6] Kalbermatten DF, Haug M, Wettstein R, Schaefer DJ, Pierer G (2005). New posterior auricular perichondrial cutaneous graft for stable reconstruction of nasal defects. Aesthetic Plast Surg.

[7] Gurunluoglu R, Shafighi M, Gardetto A, Piza-Katzer H (2003). Composite skin grafts for basal cell carcinoma defects of the nose. Aesthetic Plast Surg.

[8] Hirohi T, Yoshimura K (2003). Surgical correction of retracted nostril rim with auricular composite grafts and anchoring suspension. Aesthetic Plast Surg.

[9] Rohrer TE, Dzubow LM (1995). Conchal bowl skin grafting in nasal tip reconstruction: clinical and histologic evaluation. J Am Acad Dermatol.

[10] Keck T, Lindemann J, Kuhnemann S, Sigg O (2003). Healing of composite chondrocutaneous auricular grafts covered by skin flaps in nasal reconstructive surgery. Laryngoscope.

[11] Chandawarkar RY, Cervino AL, Wells MD (2003). Reconstruction of nasal defects using modified composite grafts. Br J Plast Surg.

